# Melioidosis in Children, Brazil, 1989–2019

**DOI:** 10.3201/eid2706.200154

**Published:** 2021-06

**Authors:** Rachel Ximenes Ribeiro Lima, Dionne Bezerra Rolim

**Affiliations:** Municipal Department of Health, Fortaleza, Brazil (R.X.R. Lima);; University of Fortaleza Medical School (R.X.R. Lima, D.B. Rolim);; Ceará State University, Fortaleza (D.B. Rolim)

**Keywords:** melioidosis, *Burkholderia pseudomallei*, pneumonia, sepsis, septic shock, children, Brazil, bacteria

## Abstract

We studied 20 confirmed or suspected cases of melioidosis in children in Ceará, Brazil, during 1989–2019. We observed a high death rate, severe signs and symptoms, and substantial environmental exposure. These data suggest that childhood melioidosis might be more severe in Brazil than in other regions.

Melioidosis, an infectious disease caused by the bacterial species *Burkholderia pseudomallei,* is associated with severe symptoms and high death rates (*1*). Although considered an emerging disease, melioidosis has little formal public health recognition (*2*). Researchers initially documented cases in Brazil in 2003 (*3*). As of 2018, Ceará, a coastal state in northeastern Brazil, has the highest incidence in South America; however, sporadic cases have been reported in other states (*4*). Although the disease predominantly affects adults with associated risk factors (*1*), the growing incidence of severe melioidosis among children and adolescents in Ceará highlights the need for clinical and epidemiologic investigations.

## The Study

We analyzed all cases of melioidosis in persons <18 years of age documented by the Ceará State Health Department during January 2005–May 2019. This state declared melioidosis a notifiable disease in 2005 (*5*), although the literature records cases from early as 1989 (*6*). We also searched for cases in the SciELO and PubMed databases using the terms “melioidosis” AND “Brazil” OR “children” published during March 2003–May 2019. We also searched the annals of Brazilian Congresses of Pediatric Infectiology from 2003–2018. In total, we identified 16 cases in the health department database (1 case was excluded because of an alternative diagnosis) and 5 in the literature (*3*,*6*). All cases were either suspected or confirmed (Table 1) (*5*,*7*).

**Table 1:** Clinical definitions in study of melioidosis in children, Brazil, 1989–2019*

Term	Definition
Suspected melioidosis	All patients with suspected melioidosis must have epidemiologic exposure at any time, recent or not, associated with >1 of the following criteria: acute febrile illness and respiratory symptoms suggestive of community pneumonia that do not improve with conventional antimicrobial treatment (β-lactam antimicrobial drugs); febrile disease that progresses with systemic inflammatory response syndrome, severe sepsis, or septic shock; prolonged fever of unknown etiology or signs and symptoms similar to tuberculosis that do not respond to tuberculosis treatment; or soft tissue infection (e.g. cutaneous ulcers/abscesses, cellulite, or fasciitis) of chronic evolution (i.e. months) with no response to conventional antimicrobial treatment (e.g. oxacillin, ampicillin associated to sulbactam, or cefalexin).
Confirmed melioidosis	All patients with confirmed melioidosis must meet laboratory (bacteriologic confirmation by microbiological culture or positive PCR) or clinical-epidemiologic criteria (exposure to the same risk situation as patients with laboratory-confirmed melioidosis). Patients with confirmed melioidosis must have signs and symptoms that are compatible with melioidosis and not attributable to a different cause.
Severe disease	Patients with severe melioidosis have clinical signs and symptoms and a high risk for death caused by pneumonia, sepsis, or septic shock.

*These criteria were defined by references (*5**,**7*).

We investigated cases using data from patient records and, when possible, from interviews with the patients and their relatives. We analyzed data on age, sex, time of symptom onset, geographic location, occupational or recreational activity involving water or soil during the 2 weeks before symptom onset, underlying conditions, signs and symptoms, laboratory and radiographic findings, clinical evolution, treatment, and clinical outcome. We used the Fisher exact test to assess the correlation between appropriate treatment using carbapenem or ceftazidime during the intensive phase of melioidosis (*8*) and survival. The study protocol was previously approved by the research ethics committees of the University of Fortaleza (Fortaleza, Brazil) (approval no. 3,094,492) and Albert Sabin Children’s Hospital (Fortaleza) (approval no. 3,194,070).

We identified 10 confirmed (including 5 before 2005: 4 in 2003, and 1 identified retrospectively in 1989) (*3*,*6*), and 10 suspected cases of melioidosis among children and adolescents. The 10 confirmed cases in persons <18 years of age account for 23.2% of the 43 confirmed cases of melioidosis in the state of Ceará as of May 2019. This proportion is substantially greater than the 5%–15% usually reported for children (*9*).

Most (9/20; 45%) patients were 10–17 years of age. The median age was 11 years for patients with confirmed cases and 9 years for those with suspected cases. For comparison, childhood melioidosis is most prevalent in children <5 years of age in Malaysia (*10*) and in children >10 years of age in Australia (*11*).

As in previous studies (*2*,*12*), most (13/20; 65%) patients in this sample were male. Illnesses occurred most frequently during the rainy season (i.e. February–May), accounting for 65% (13/20) of all cases and 70% (7/10) of confirmed cases. This trend resembles the results of a study in Australia (*11*) and reinforces the association between heavy rainfalls and exposure to *B. pseudomallei*. Most (19/20; 95%) patients had environmental exposure during the 14 days before symptom onset (Table 2). Outdoor recreational behavior is common among children in Brazil, especially in the tropics. For example, when intense warm showers interrupt the extended droughts of northeastern Brazil, children often bathe and play in waterfalls, rivers, and dams. This might partially account for the high prevalence of melioidosis among children, especially older children and boys, in this region.

**Table 2:** Clinical and epidemiologic characteristics of children with melioidosis, Brazil, 1989–2019*

Pt	Age, y/sex	City	Rainy season†	Potential exposures‡	Pneumonia	Sepsis	Septic shock	Diagnostic results	Timely treatment#	Outcome (time to death)¶
1	0.25/M	Fortaleza	No	Mother lived in rural area during pregnancy§	No	No	No	*Pseudomonas pseudomallei* in cerebrospinal fluid	Yes	Survived
2	15/M	Tejuçuoca	Yes	Swam in river	Yes	Yes	Yes	No test, met clinical epidemiologic criteria	No	Death (40 h)
3	14/F	Tejuçuoca	Yes	Swam in river	Yes	Yes	Yes	*Burkholderia pseudomallei*	No	Death (90 h)
4	10/M	Tejuçuoca	Yes	Swam in river	Yes	Yes	Yes	*B. pseudomallei*	No	Death (6 d)
5	12/F	Tejuçuoca	Yes	Swam in river	Yes	Yes	Yes	*B. pseudomallei*	Yes	Survived
6	17/M	Fortaleza	Yes	Bathed in river/waterfall	Yes	Yes	Yes	*B. pseudomallei*	Yes	Death (10 d)
7	3/M	São João do Jaguaribe	Yes	Swam in river	Yes	Yes	Yes	*B. pseudomallei*	Yes	Death (28 d)
8	13/F	Ipu	No	Bathed in waterfalls	Yes	Yes	Yes	*B. pseudomallei*	No	Death (10 d)
9	3/F	Granja	Yes	Swam in river, bathed in waterfalls	Yes	Yes	No	No test, met clinical epidemiologic criteria	Yes	Survived
10	6/M	Fortaleza	No	Swam in river, bathed in waterfalls	Yes	Yes	Yes	*B. pseudomallei* in bronchoalveolar lavage, met clinical epidemiologic criteria	Yes	Survived
11	6/M	Limoeiro do Norte	No	Swam in river, bathed in waterfalls, fished, drank contaminated water	No	No	No	Negative	Yes	Survived
12	9/F	Pacatuba	No	Swam in river, bathed in waterfalls	Yes	Yes	Yes	Negative	Yes	Death (5 d)
13	13/M	Guaiúba	No	Swam in river, bathed in waterfalls, fished	Yes	Yes	Yes	*B. cepacea* in oropharyngeal swab sample	Yes	Survived
14	1/F	Fortaleza	No	Swam in untreated pool	Yes	Yes	Yes	Negative	Yes	Survived
15	6/M	Canindé	Yes	Swam in river/dams, fished	Yes	Yes	Yes	Negative	No	Death (8 d)
16	3/M	Fortaleza	Yes	Swam in lake/ played with soil	Yes	Yes	Yes	Negative	Yes	Survived
17	9/F	Canindé	Yes	Swam in river/dams, fished	Yes	Yes	Yes	Negative	Yes	Survived
18	11/M	Orós	Yes	Swam in river, fished	Yes	Yes	Yes	Negative	Yes	Survived
19	14/M	Trairí	Yes	Swam in river/dams, fished	Yes	Yes	Yes	Negative	No	Death (4 d)
20	9/M	Trairí	Yes	Swam in river/dams, fished	Yes	Yes	Yes	Negative	Yes	Survived

*Cases 1–10 were confirmed according to diagnostic criteria (5,7); cases 11–20 were suspected. Pt, patient. †Rainy season in Ceará, Brazil is February–May. ‡During 14 d before symptom onset. ¶From symptom onset.  §Potential vertical transmission. #As defined in (*8*).

The most frequent clinical manifestations were sepsis (18/20; 90%), pneumonia (18/20; 90%), and septic shock (17/20; 85%) (Table 2). Among confirmed cases, 90% (9/10) of patients had sepsis and pneumonia and 80% (8/10) had septic shock. Among suspected cases, 90% (9/10) of patients had pneumonia, sepsis, and septic shock. Studies in Malaysia have reported similar figures (*10*); however, the main manifestations among children are skin lesions in Australia and infectious parotitis in Cambodia (*13*,*14*). Although the methods used by these studies differ, they suggest that children in Ceará might have more severe clinical manifestations of melioidosis.

Two patients had meningitis, accounting for 20% (2/10) of confirmed cases and 10% (2/20) of total cases; however, a study in Australia observed neuromelioidosis in 3% of pediatric patients (*15*). These findings might indicate either a greater proportion of neurologic involvement or substantial underreporting of less severe manifestations among children with melioidosis in Brazil.

In total, 45% (9/20) of patients died: 60% (6/10) of patients with confirmed cases and 30% (3/10) of those with suspected cases. Childhood melioidosis is associated with a death rate of 35% globally (*9*), although in Australia the rate is reported to be 7% (*13*). In Cambodia, 16.4% of patients die, including up to 71% of patients with bacteremia (*14*). Our findings, which include high prevalence of sepsis and septic shock, 2 cases of severe neurologic involvement, and high death rates, warrant further investigation.

We found that appropriate, timely treatment for melioidosis (*8*) was significantly associated with survival among 20 patients (p<0.01). Thus, physicians should consider empirical treatment for suspected melioidosis in patients in areas to which the disease is endemic, especially if the initial treatment was unsuccessful. We did not find a significant association between proper treatment and survival among patients with confirmed (p = 0.08) and suspected cases (p = 0.07) of melioidosis, possibly because of small sample size.

## Conclusion

We describe a high prevalence, death rate, and severity of childhood melioidosis in Brazil. The high death rate and clinical severity might be partially explained by underreporting of mild cases, but the frequent environmental exposures of children in this region warrant further research. These findings emphasize the need for melioidosis awareness among healthcare providers and laboratory professionals. Physicians should consider melioidosis as a differential diagnosis; improved awareness might reduce underreporting and optimize the quality of epidemiologic data. Physicians also should consider empirical treatment in patients who have clinical manifestations compatible with the disease and whose prognosis is compromised by clinical severity.
